# Body size estimation in women with anorexia nervosa and healthy controls using 3D avatars

**DOI:** 10.1038/s41598-017-15339-z

**Published:** 2017-11-17

**Authors:** Katri K. Cornelissen, Kristofor McCarty, Piers L. Cornelissen, Martin J. Tovée

**Affiliations:** 1 Department of Psychology, Northumbria University, Newcastle Upon Tyne, NE1 8ST United Kingdom; 2 School of Psychology, Lincoln University, Lincoln, LN6 7TS United Kingdom

## Abstract

A core feature of anorexia nervosa is an over-estimation of body size. However, quantifying this over-estimation has been problematic as existing methodologies introduce a series of artefacts and inaccuracies in the stimuli used for judgements of body size. To overcome these problems, we have: (i) taken 3D scans of 15 women who have symptoms of anorexia (referred to henceforth as anorexia spectrum disorders, ANSD) and 15 healthy control women, (ii) used a 3D modelling package to build avatars from the scans, (iii) manipulated the body shapes of these avatars to reflect biometrically accurate, continuous changes in body mass index (BMI), (iv) used these personalized avatars as stimuli to allow the women to estimate their body size. The results show that women who are currently receiving treatment for ANSD show an over-estimation of body size which rapidly increases as their own BMI increases. By contrast, the women acting as healthy controls can accurately estimate their body size irrespective of their own BMI. This study demonstrates the viability of combining 3D scanning and CGI techniques to create personalised realistic avatars of individual patients to directly assess their body image perception.

## Introduction

Anorexia nervosa (AN) is a serious psychological and physiological condition, which occurs predominantly in the female population. Current therapeutic regimes have only a limited success in treating this condition^1^, where the long-term mortality rate has been estimated to be as high as 10%^2^. To be able to treat this condition more effectively, we need a better understanding of its central features. Diagnostic criteria for AN include a distorted evaluation of personal body size^3^, and this is also a key element of psychological models of the disorder^4,5^. This distorted body image motivates weight loss behaviours that become more entrenched and resistant to change over time, and culminate in a dangerously low body-mass index^6,7^. Body image distortion has been shown to be one of the most persistent of all the eating disorder symptoms, the severity of which seems to predict the long-term outcome for patients^5,8^. Furthermore, persistence of body image distortion has been shown to predict the rate of relapse^8,9^ which has been estimated to be as high as 35%^10^. While there is evidence to suggest that women with AN under-estimate their body size^11^, or even show performance in size estimation tasks equivalent to controls^11,12^, most studies have found that patients with AN overestimate their body size^9,13–15^.

## Measuring body image distortion: the need for realistic stimuli

A variety of methods has been used to estimate the perceptual dimension of body image distortion, starting from image marking procedures^16^ and moveable calliper techniques^9^, through body-distorting mirrors^17^ and silhouette methods^18^ to distorting photograph and video techniques^19–22^. In many early studies, figure scales were used where participants were asked to identify which figure they believed to be closest to their own. However, their use has largely fallen out of favour owing to the lack of validated psychometric properties^23^. Methods that are more recent include applying body part morphs to photographs of individuals^9,15^ and the whole-body video distorting technique (VDT)^24–26^. The distinction between whole body and body part methods, which has persisted throughout the literature, gave rise to concern about how comparable the results were from the two methodologies. Slade was first to point out that outcomes, reliability and sensitivity were often different, such that body part methods often gave rise to overestimates and whole body methods frequently led to underestimation^27^. However, a formal meta–analytic comparison between these two approaches to body size estimation showed convincingly that both methodologies showed overestimation by participants with anorexia nervosa (AN)^28^. While the average effect size was smaller for the whole body method, it showed far greater consistency than the body part method across studies.

The principal behind most of the whole-body measurement methods has been to present participants with images of a body that have been expanded or contracted in the horizontal dimension, to simulate changes in adiposity. This has been achieved in a variety of ways. In the distorting photograph technique, a slide of the person’s body is projected on to a screen through a variable anamorphic lens. Participants adjust the width of the optically distorted image on the screen until it matches the size they believe themselves to be^20^, and the percentage distortion of the image is recorded. This may be negative (underestimation), zero (accurate perception) or positive (over-estimation). Equivalent methods using video (i.e. the video distorting technique, VDT) have been developed^24^, and these have even been extended to a life size screen distortion method^29^. A serious problem with whole body image manipulations such as the VDT, which compress/expand the horizontal axis of the stimulus image, is that they amount to a ‘multiplicative’ model for representing body weight change – i.e. the horizontal width of the stimulus image is scaled multiplicatively^30^. While this changes the width of the body consistent with changing adiposity^31^, it fails to capture other structural changes in the abdomen, chest and limbs, and it comes at the expensive of introducing systematic distortions of body shape which do not occur in reality^32^. For example, the width of the shoulders and the hips in particular change inappropriately. Moreover, features such as the width of the gap between the thighs, which normally would be expected to reduce with increasing adiposity, actually increases with increasing image expansion in the VDT. Finally, waist hip ratio (WHR) in UK females should show a monotonically increasing, decelerating relationship with BMI, yet the multiplicative model of fat deposition completely fails to capture this relationship^30^.

## A standard CGI model as stimulus or personalized 3D avatars?

An alternative strategy has asked participants to estimate their body size by comparing themselves to stimuli which were created by manipulating the BMI dependent body shape changes of the same ‘standard’ 3D model^32–35^. The use of CGI modelling techniques allows an accurate simulation of adipose loss or deposition on the female body. Additionally, it allows: (a) the identity of the person in the image is clearly maintained over a wide BMI range; (b) the images to be calibrated for BMI, and therefore a participant’s body size estimate in BMI units can be compared with their actual body size in BMI units; (c) the 3D rendered stimulus images are high definition and photorealistic. These images have been used in yes-no paradigms, in which a participant indicates over a number of trials whether the image on any one trial is thinner or fatter than themselves. The participant’s estimate of their own body size can then be computed from the psychometric function which plots the proportion of ‘fatter’ responses (y-axis) as a function of the BMI (x-axis) of the stimuli^32–35^.

**Figure 1:**
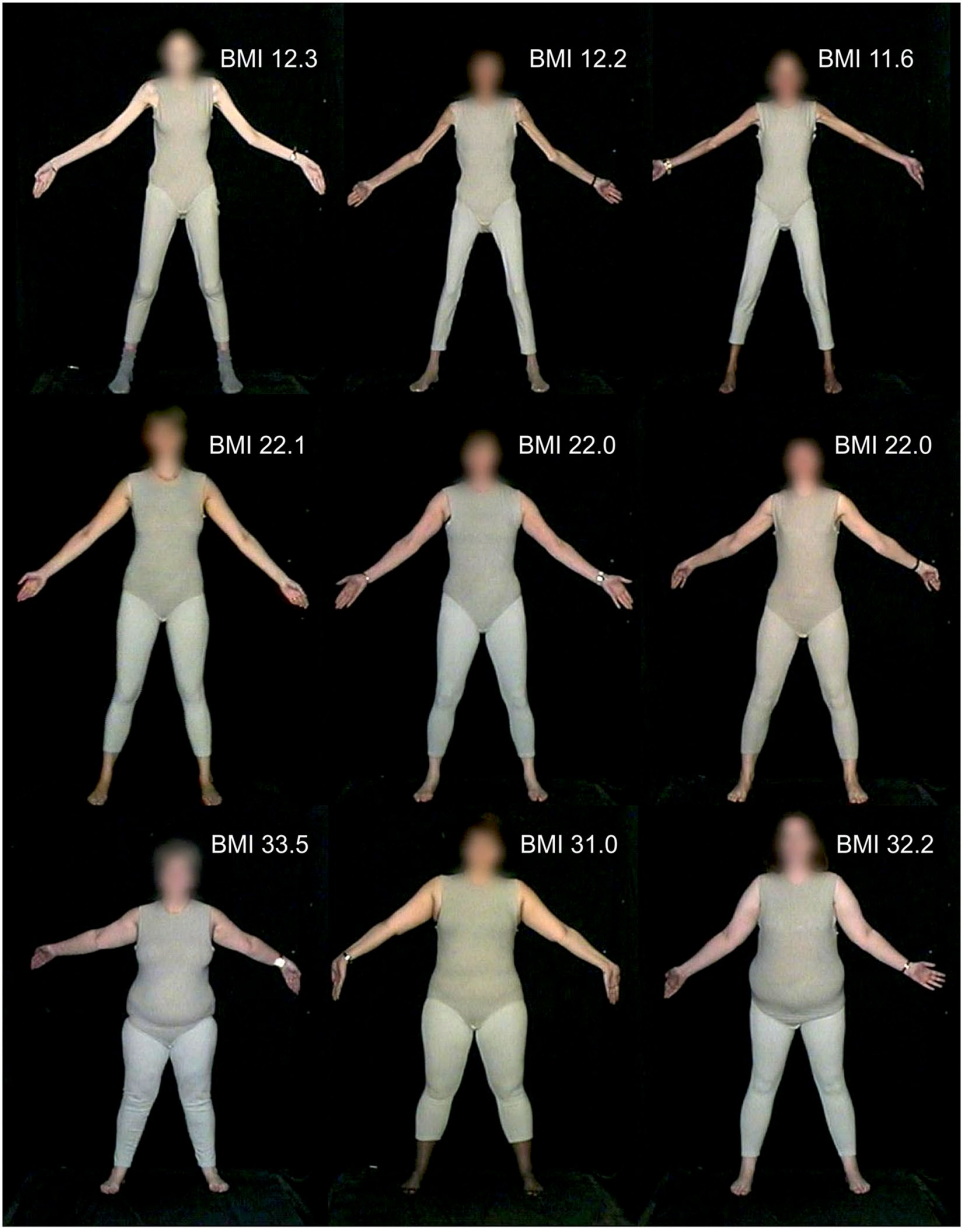
Illustration of real body shape variation, across each row, in individuals with approximately the same BMI (images drawn from the image library described in Tovée *et al*.^56^).

However, there are two potential problems with this strategy. The first is that it ignores individual variation in underlying body shape across different observers (see Fig. 1 for illustration). The standard body shape used in a 3D model may be a good fit for some body shapes, but not so good for others. Therefore, this immediately creates a participant dependent source of variability in the experiment that cannot be controlled easily. Secondly, asking a participant to judge themselves against another individual, is one step further removed than would ideally be the ecologically valid case – i.e. what we would really like to do is to create a situation equivalent to looking in the mirror. Therefore, we must assume that there is likely to be an additional cognitive load, and therefore an uncontrolled source of variability, in asking participants to map their belief about their own body shape and size on to the image of a third party. Together, we will refer henceforth to these potential confounds as the body schema to stimulus mapping problem. Furthermore, as far as AN participants are concerned, different body parts can make a great impact on an individual’s perception of their body size. It has even been suggested that the overestimation of body size may be due to overestimation of certain body areas^13,15,36,37^, particularly the hips and thighs^38,39^.

To move towards a solution to these problems, in this study we used a Size Stream 3D body scanner to capture a 3D template which accurately represents an individual participant’s body shape. This template was then imported to a 3D modelling environment to fit the base model to the template, effectively generating a high-resolution clone of an individual’s body. Finally, the whole-body shape morphs in the modelling environment were manipulated to systematically increase or decrease the BMI of the cloned participant. Each participant then estimated their actual body size (indexed by estimated BMI) in two ways: (i) using a standard CGI model together with a yes-no task, and (ii) using their avatar model in combination with a method of adjustment task. If the outcomes from the two methods failed to converge on the same result, this would suggest that there is an important body schema to stimulus mapping problem with body size estimation tasks, and that further research is needed to develop ecologically valid stimulus sets.

## Perceptual biases in judging body size: a test of two hypotheses

An additional, important complication in judging body size is contraction bias^32,33^. We make judgements about complex stimuli, such as bodies, by reference to a template based on the average of all the examples of that class of stimuli that we have seen - our “visual diet”^40,41^. Errors in judging body size arise as a direct result of this way of estimating magnitude. When one uses a standard reference or template for a particular kind of object against which to estimate the size of other examples of that object, a form of visual bias called contraction bias occurs, which is perfectly normal. The estimate is most accurate when a given object is of a similar size to the reference, but becomes increasingly inaccurate as the magnitude of the difference between the reference and the object increases. As a result, the observer estimates that the object is closer in size to the reference than it actually is. Thus, an object smaller in size than the reference will be over-estimated and an object larger will be under-estimated. Contraction bias therefore predicts that individuals judging very thin bodies will over-estimate their body size. This would be true of women with AN but also women without AN, but with a low BMI. Therefore, the body size over-estimation found in women with AN^9,13–15^ could be unrelated to their psychological condition and may simply be a by-product of contraction bias when estimating low BMI bodies. If this hypothesis is correct, and the over-estimation of body size in women with AN is purely due to a normal perceptual bias, then as women with AN increase their BMI, the accuracy of their judgements should *improve*. Specifically, the regression of estimated BMI on actual BMI should be the same for women with AN and healthy controls, and should be consistent with contraction bias; i.e. a slope < 1. Alternatively, it is entirely possible that psychological factors represent a stronger driving force behind body size over-estimation than perceptual factors. If so, an individual’s body size (as indexed by BMI) is known to be strongly correlated with body dissatisfaction^42–44^. Women with AN who have achieved a very low BMI might be expected to have relatively low body size concerns, but during the recovery process as their weight increases, their body size concerns would rise in parallel. Therefore, an alternative outcome for women with AN is that as their weight increases, there is a rapid *rise* in the degree of body size over-estimation reflecting their accelerating concerns about body shape and weight. Statistically speaking, in this situation the regression of estimated BMI on actual BMI should show evidence of contraction bias for healthy controls, but should show a slope > 1 for women with AN.

To distinguish between these two hypotheses, we used both standard CGI stimuli, as well as our new measurement technique with personalised 3D scanned bodies, which avoid the body schema to stimulus mapping problem. We recruited a group of women who have all had a diagnosis of AN and who are still in treatment, but who now show significant variation in their BMI. These women should more correctly be referred to as suffering from anorexia spectrum disorders (ANSD)^32,35^. By deliberately taking advantage of individual differences in this way, we can determine whether the accuracy of body size estimation increases or decreases as the BMI of ANSD participants varies and therefore determine the relative importance of psychological factors and perceptual bias.

## Experiment 1 Methods

The experimental procedures and methods for participant recruitment for this study were approved by: the local ethics committee at Northumbria University; the Beating Eating Disorders Organisation (BEAT) and the Northern Initiative on Women and Eating (NIWE) Organisation. All experiments were performed in accordance with relevant guidelines and regulations set out by these organizations and all participants gave their informed consent to take part in this study.

### Participants

Using a standard CGI model stimulus together with a yes-no task, Cornelissen *et al*.^32^ found that the slopes of the regression of estimated BMI on actual BMI were 1.39 and 0.82 for women with ANSD and healthy controls respectively. With respect to veridical performance in this task, which would be revealed by a regression slope of 1 and an intercept of zero, the observed values represent a 39% increase for women with ANSD and an 18% decrease for healthy controls. Therefore, we powered a sample size calculation for the current study based on the healthy controls since this smaller value should be harder to detect. Taking performance on the psychometric tasks into account, we used PROC POWER in SAS v9.4 (SAS Institute, North Carolina, USA) to show that 13 participants should suffice to demonstrate a regression slope less than 1 at α = 0.05 with a power (1 - β) of 0.9. To offset attrition in participant numbers and/or unexpected sources of variability, we therefore recruited 15 female participants from the population of undergraduate students at Newcastle and Northumbria Universities and from the general population in and around the Newcastle upon Tyne area, all of whom consented to take part in the study as controls. No control participants had a history of eating disorders. In addition, we recruited 15 female participants into the study all of whom had a formal diagnosis of anorexia nervosa according to DSM-IV-R or DSM-5^3,45^ and who were still receiving treatment at the time of testing, but whose BMI ranged more widely than is typical under this strict definition (hence referring to these individuals as having ANSD). Table 1 shows the characteristics of these two participant groups.

**Table 1:** Characteristics of the participants.

	ANSD (*n* = 15)	CON (*n* = 15)	ANSD vs CON
	*M (SD)*	*M (SD)*	*p*
Chronological age (years)	24.02 (3.71)	29.73 (5.31)	0.02
BMI	18.44 (2.88)	23.67 (4.32)	0.005
Over-estimation (PSE-BMI)	5.45 (4.74)	0.27 (2.57)	0.001
BSQ	67.53 (16.25)	51.80 (22.54)	0.04
BDI	26.40 (14.95)	10.13 (9.81)	0.01
EDE-Q	3.94 (1.20)	2.18 (1.24)	0.005
EDE-Q res	3.51 (1.54)	2.24 (1.38)	0.04
EDE-Q wc	3.47 (1.56)	1.23 (1.30)	0.002
EDE-Q sc	4.09 (1.33)	2.33 (1.41)	0.01
EDE-Q eat	4.68 (1.05)	2.90 (1.77)	0.01

*Note*. ANSD: participants with anorexia nervosa who were being treated at the time of testing. CON: healthy, non eating-disordered controls. BMI = Body Mass Index. PSE = Point of Subjective Equality. BSQ = Body Shape Questionnaire. BDI = Beck Depression Inventory. EDE-Q = Eating Disorder Examination Questionnaire global score. EDE-Q res = Eating Disorder Examination Questionnaire eating restraint subscale. EDE-Q wc = Eating Disorder Examination Questionnaire weight concern subscale. EDE-Q sc = Eating Disorder Examination Questionnaire body shape concern subscale. EDE-Q eat = Eating Disorder Examination Questionnaire eating concern subscale.

### Psychometric and biometric measurements

To assess participants’ attitudes to body shape, weight and eating we used the 16-item Body Shape Questionnaire (BSQ) (range 0–96^46^) which indexes the degree of preoccupation and negative attitude toward body weight and body shape. In addition, we used the Eating Disorders Examination Questionnaire (EDE-Q), which is a self-report version of the Eating Disorder Examination (EDE) structured interview^47^. This is commonly used as a screening questionnaire for eating disordered behaviour and has been normed for young women and undergraduates^48,49^. The questionnaire contains four subscales reflecting the severity of aspects of the psychopathology of eating disorders: (i) the Restraint (EDE-Q res) subscale investigates the restrictive nature of eating behaviour; (ii) the Eating Concern (EDE-Q eat) subscale measures preoccupation with food and social eating; (iii) the Shape Concern (EDE-Q sc) subscale investigates dissatisfaction with body shape and (iv) the Weight Concern (EDE-Q wc) subscale assesses dissatisfaction with body weight. The EDE-Q (range 0–6) also measures overall disordered eating behaviour. Furthermore, it provides frequency data on key behavioural features of eating disorders. We also used the Beck Depression Inventory (BDI) (range 0–63^50^) that measures participants’ level of depression, and we calculated the participants’ body mass index (BMI) from their weight obtained with a set of calibrated scales and their height obtained with a stadiometer.

### Stimulus image preparation

#### Standard model

To create stimulus images which correctly represent how an individual body shape changes as a function of changing BMI, we used computer-generated imagery (CGI) methods (DAZ 3D Studio, v4.8) to create graded 3D images of a standard model where: i) the identity of the person in the image is clearly maintained over a wide BMI range; ii) the body shape changes at different BMI levels are extremely realistic and iii) the 3D rendered stimulus images are high definition and photorealistic (see^32,51^ for further technical details). In addition, we made precise estimates of the BMI of the 3D model in our stimulus images. To achieve this, we used the Health Survey for England^52,53^ datasets to create calibration curves between waist and hip circumferences and height derived from ~3000 women in the UK, aged between 18 and 45. Because our CGI model exists in an appropriately scaled 3D world, having set the height of our models (1.6 m) we can therefore measure their waist and hip circumferences, and compare these with our HSE calibration curves to compute their BMI.

#### Avatars

CGI avatars were created as follows. First, each participant had a 3D body scan. In a private booth, participants wore underwear only while their body shape was captured using a Size Stream Body Scanner (using scanner software v4.4). This device comprises a set of 14 infra-red depth sensors arranged around the body, each individually fixed to the rigid frame of the booth. Once in the scanner, participants adopted a standard pose while holding hand-rails to steady themselves. They were asked to exhale midway and not to move for ten seconds while the scan was completed. The circumferential accuracy of the system, using a test cylinder ~ 880mm tall, is less than +/−5 mm.

The data generated by the scan, a large point cloud, were immediately stored off-line by the Size Stream Studio software, converted into a 30k polygon mesh and this in turn was read into DAZ 3D Studio (v4.8). We used the morphing tools in this modelling environment to restructure the Genesis 2 female base model to have the same shape as the body scan mesh, with the same height, and same bodily proportions. When the modelling was finished, we used the DAZ Measurement Metrics (v1.1) tools to measure the following circumferences from the avatar model: bust, under-bust, waist, hips, upper arm and mid-thigh and compared them to the equivalent measures from the scanned data provided by the Size Stream Scanner software. Our criteria for an adequate model fit to the scan data were that: (i) these key measurements from Measurement Metrics should be within +/−3 percent of the scan data; (ii) there should be minimal distance between the scan mesh surface and the model surface throughout the entire model; (iii) there should be a good qualitative fit between the scan data and the avatar model – i.e. the avatar should obviously look like body scan mesh. Once these criteria were met, we applied a standard skin to all avatars, a standard sports bikini, and the same background and lighting. We then used the 3Delight render engine (v1.12) to generate a sequence of 120 images from the Genesis 2 avatar in order to represent the body shape changes that occur in the BMI range from ~13 to 45.

### Psychophysical measurement procedures

#### Yes-no task with standard model

In the yes-no task, participants were presented with a randomized sequence of images of the standard female body model. Across the image set, BMI varied continuously from 12.5 to 44.5. On each trial of the task, one image was presented and participants were required to decide whether the body depicted was larger or smaller than they were. Stimuli were presented on a 19” flat panel LCD screen (1280w × 1024 h pixel native resolution, 32-bit colour depth) for as long as it took participants to make a decision. At the standard viewing distance of ~60 cm, the image frame containing the female body subtended ~26° vertically and ~8° degrees horizontally. Each participant first judged 7 images covering the whole BMI range (from 12.5 to 44.5 in equal BMI steps) presented in 2 separate blocks. Each stimulus image appeared 10 times in each block, and the order of presentation was randomized. Based on the responses from each block, the participants’ point of subjective equality or PSE (the BMI they believe themselves to be) was calculated automatically by fitting a cumulative normal distribution. These two values were then averaged to give an initial estimate of the participant’s PSE. On the basis of this initial estimate, the program presented a further set of 21 images (spread over a range of 5 BMI units centred on the participant’s initial PSE, at a spacing of 0.25 units per image) for the participants to judge. Each image was presented 10 times in randomized order. This final set of judgements allowed us to plot the full psychometric function (i.e. the proportion of ‘larger’ responses on the y-axis as a function of stimulus BMI on the x-axis) and use probit analysis off-line to calculate a definitive estimate of PSE as well as the difference limen or DL (that is how sensitive participants are to changes in BMI).

#### Method of adjustment tasks with avatar

**Figure 2:**
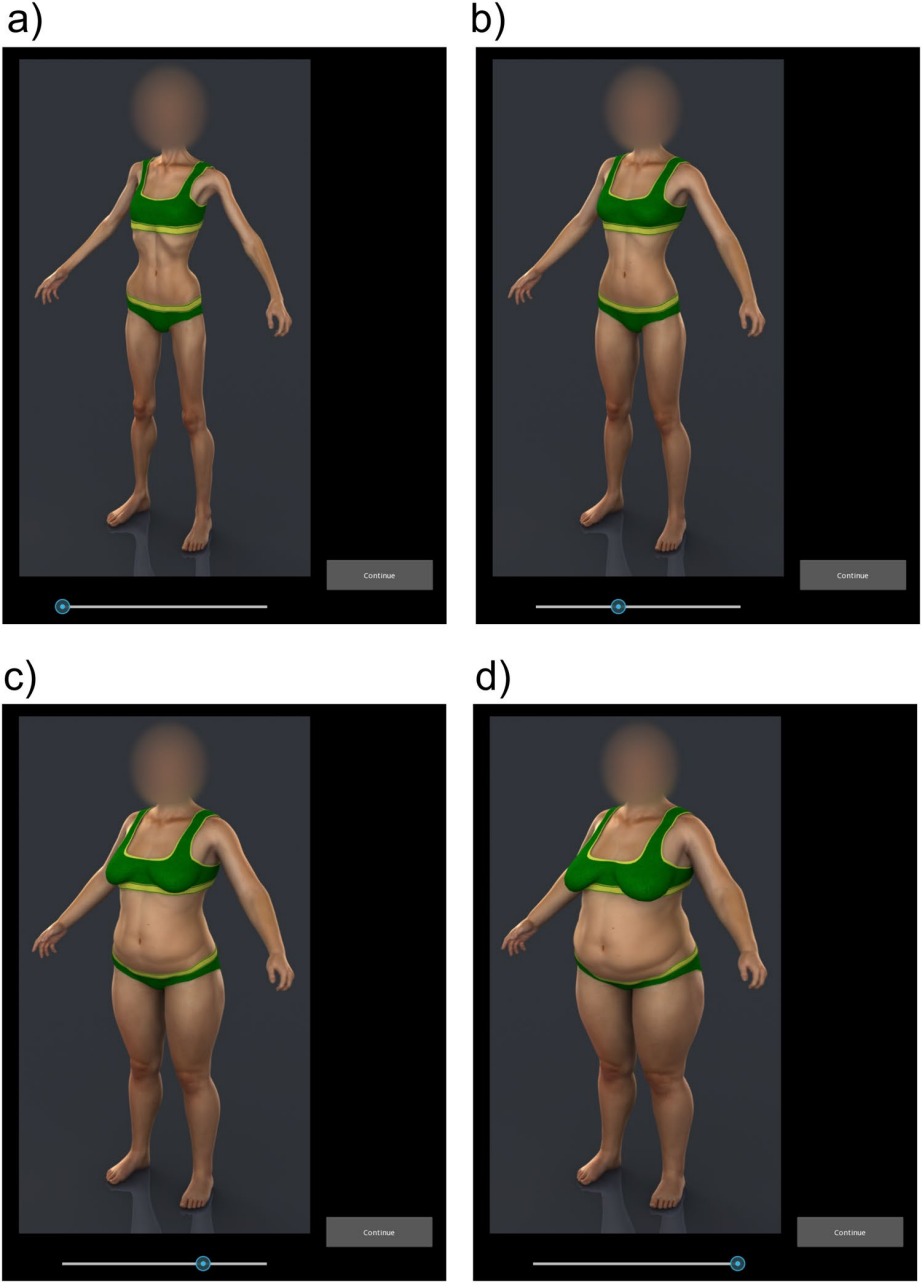
Body shape changes for an anonymized, bespoke avatar as the slider control is moved from left to right through screenshots **a**,**b**,**c** & **d**.

Participants used the method of adjustment to estimate their body size under each of three task conditions whose order was randomized across participants. Participants had to decide: the size and shape they believed themselves to have while viewing the whole avatar (WHOLE); the size and shape they believed themselves to have when they could only see the avatar from the waist down to the legs (LEGS); the size and shape they believed themselves to have when they could only see the avatar from the waist up to the head (TORSO). For each condition, participants carried out 20 trials using the same display setup as for the yes-no task. On each of these 20 trials, the participant’s avatar appeared on screen with the face blurred. Beneath the avatar was a slider control. The participant was asked to click on the slider control to move it from side to side. When the slider moved leftwards the BMI of the avatar reduced smoothly to a minimum of ~13 and increased to a maximum of ~45 when the slider moved rightward. The participant had to decide which image in this continuum fitted best the criterion for the particular experimental condition, and then press a radio button on screen that allowed the stimulus PC to log their response and initiate the next trial. At the start of each trial, the BMI of the avatar was set randomly to either its minimum, with the slider appearing at the leftmost extreme of its range of movement, or the maximum BMI, with the slider appearing at the rightmost extreme of its range of movement. Figure 2 illustrates four sequential frames from the method of adjustment task. Finally, we note that the order of presentation of the yes-no and method of adjustment tasks was counterbalanced across participants.

### Timeline for Task Administration

Each participant made two visits. On the first visit, the participants filled in the psychometric scales and were scanned. We then created their avatar and rendered the stimuli. Each participant then returned to carry out the psychophysical task within a week of being scanned.

### Data Availability

The datasets generated during and/or analysed during the current study are available from the corresponding author on reasonable request.

## Experiment 1 Results

### Psychometric Task Reliability

The responses to the psychometric questionnaires across the sample showed good internal reliability scores. For the Beck Depression Inventory (BDI), Body Shape Questionnaire (BSQ) and Eating Disorder Examination Questionnaire (EDE-Q), Cronbach’s alpha was: 0.90, 0.96 and 0.90 respectively.

### Univariate statistics

Table 1 shows the means and standard deviations (SD) for the participant characteristics, separated according to whether they belong to the ANSD or control (CON) group. The right most columns of Table 1 show the output of pairwise comparisons of these group means, adjusted for multiple comparisons, using the permutation method in PROC MULTEST (SAS v9.4, SAS Institute, North Carolina, US). Consistent with previous literature we found that when compared to the controls, ANSD participants had statistically significantly lower BMIs. In addition, ANSD participants had elevated concerns about body shape, eating behaviour and body weight (BSQ, EDE-Q and EDE-Q sub-scores), greater tendency towards depression and significantly greater over-estimation of body size (OE).

### Whole sample analysis: psychometric measures

Ultimately, we wanted to model the relationships between participants’ BMI estimated under the two experimental manipulations (i.e. yes-no versus method of adjustment), GROUP assignment (i.e. ANSD versus CON), using actual BMI as a predictor variable. In addition, we wanted to control for any influence of AGE and the psychometric variables (BDI, BSQ and EDE-Q). In order to avoid the possibility of introducing substantial variance inflation into the models, we first checked for evidence of co-linearity amongst the psychometric variables. Table 2 shows the Pearson correlation matrix for the psychometric measures and BMI across all 30 participants. All the correlations between the psychometric measures were statistically significant at p < 0.001

**Table 2:** Pearson correlations between psychometric measures and BMI from the whole sample.

	BSQ	BDI	EDE-Q	EDE-Q res	EDE-Q wc	EDE-Q sc	EDE-Q eat
BSQ	—						
BDI	0.68**	—					
EDE-Q	0.86**	0.71**	—				
EDE-Q res	0.60**	0.51**	0.80**	—			
EDE-Q wc	0.75**	0.70**	0.92**	0.62**	—		
EDE-Q sc	0.79**	0.65**	0.92**	0.62**	0.80**	—	
EDE-Q eat	0.91**	0.69**	0.94**	0.64**	0.83**	0.87**	—
BMI	0.12	−0.33*	−0.18	−0.09	−0.33*	−0.09	−0.12

^∗^p <= 0.05, **p <0.001.

*Note*. BSQ = Body Shape Questionnaire. BDI = Beck Depression Inventory. EDE-Q = Eating Disorder Examination Questionnaire global score. EDE-Q res = Eating Disorder Examination Questionnaire eating restraint subscale. EDE-Q wc = Eating Disorder Examination Questionnaire weight concern subscale. EDE-Q sc = Eating Disorder Examination Questionnaire body shape concern subscale. EDE-Q eat = Eating Disorder Examination Questionnaire eating concern subscale.

Given these substantial correlations, we therefore used PROC FACTOR in SAS v9.4 (SAS Institute, North Carolina, USA) to carry out a principal components analysis with rotation in order to identify the significant latent variable(s) in the psychometric data. (NB we used the EDE-Q sub-scores and excluded the global EDE-Q measure, in order to avoid repetition). We then used the factor scores from these latent variable(s) in our statistical models. The Kaiser-Meyer-Olkin (KMO) measure of sampling adequacy (which indicates the degree of diffusion in the pattern of correlations) was 0.89 suggesting an acceptable sample. One factor had an Eigen value greater than Kaiser’s criterion of 1 (i.e. 4.58) which explained 76% of the variance. The scree plot showed an inflexion, i.e. Cattel’s criterion which also justified retaining just the one factor. The residuals were all small, and the overall root mean square off-diagonal residual was 0.060, indicating that the factor structure explained most of the correlations. The factor loadings for BDI, BSQ, EDE-Q res, EDE-Q wc, EDE-Q sc and EDE-Q eat were: 0.80, 0.91, 0.75, 0.90, 0.91 and 0.95 respectively. This latent variable, referred to henceforth as PSYCH, represents a combination of the attitudes thought to contribute to body size disturbance: disturbed attitudes to eating, weight and shape, and tendency towards depression.

### Whole sample analysis: over-estimation between experimental conditions

Table 3 shows the Pearson correlations between the psychometric factor PSYCH, chronological age and over-estimation of body size (i.e. estimated BMI minus actual BMI) computed from the three conditions of the method of adjustment task (i.e. the size and shape participants believed themselves to have while viewing: (i) the whole avatar, WHOLE, (ii) from the waist down to the legs, LEGS, (iii) from the waist up to the head, TORSO) as well as from the yes-no method of constant stimuli.

**Table 3:** Pearson correlations between PSYCH, chronological age and body size over-estimation across all participants.

			PSYCH	AGE	OVER-ESTIMATION Method of Adjustment			
					WHOLE	LEG	TORSO	AVERAGE
		AGE	−0.28	—				
OVER-ESTIMATION	Method of Adjustment	WHOLE	0.27	−0.22	—			
		LEG	0.36*	−0.04	0.78***	—		
		TORSO	0.15	−0.33	0.89***	0.68***	—	
		AVERAGE	0.28	−0.21	0.96***	0.89***	0.93***	—
	Yes-No	PSE-BMI	0.47**	−0.32	0.78***	0.62***	0.61***	0.72***

*p < 0.05; **p < 0.01; ***p < 0.001.

The striking finding illustrated in Table 3 is that body size over-estimation based on viewing either the whole body, or just from waist down, or just from waist up, produced very similar outcomes. This strongly suggested that we should treat the WHOLE, LEG and TORSO estimates as equivalent to each other [respective means and SEs: 22.47 (0.95), 22.99 (1.02), 22.40 (0.98)], thereby justifying computing an average of these scores per participant, henceforth referred to as AVERAGE. Finally, we confirmed that PSYCH was not correlated with participants’ BMI (r = −0.16, p > 0.1), justifying using both of these as independent explanatory variables in the multivariate analysis.

### Group Analysis

**Figure 3:**
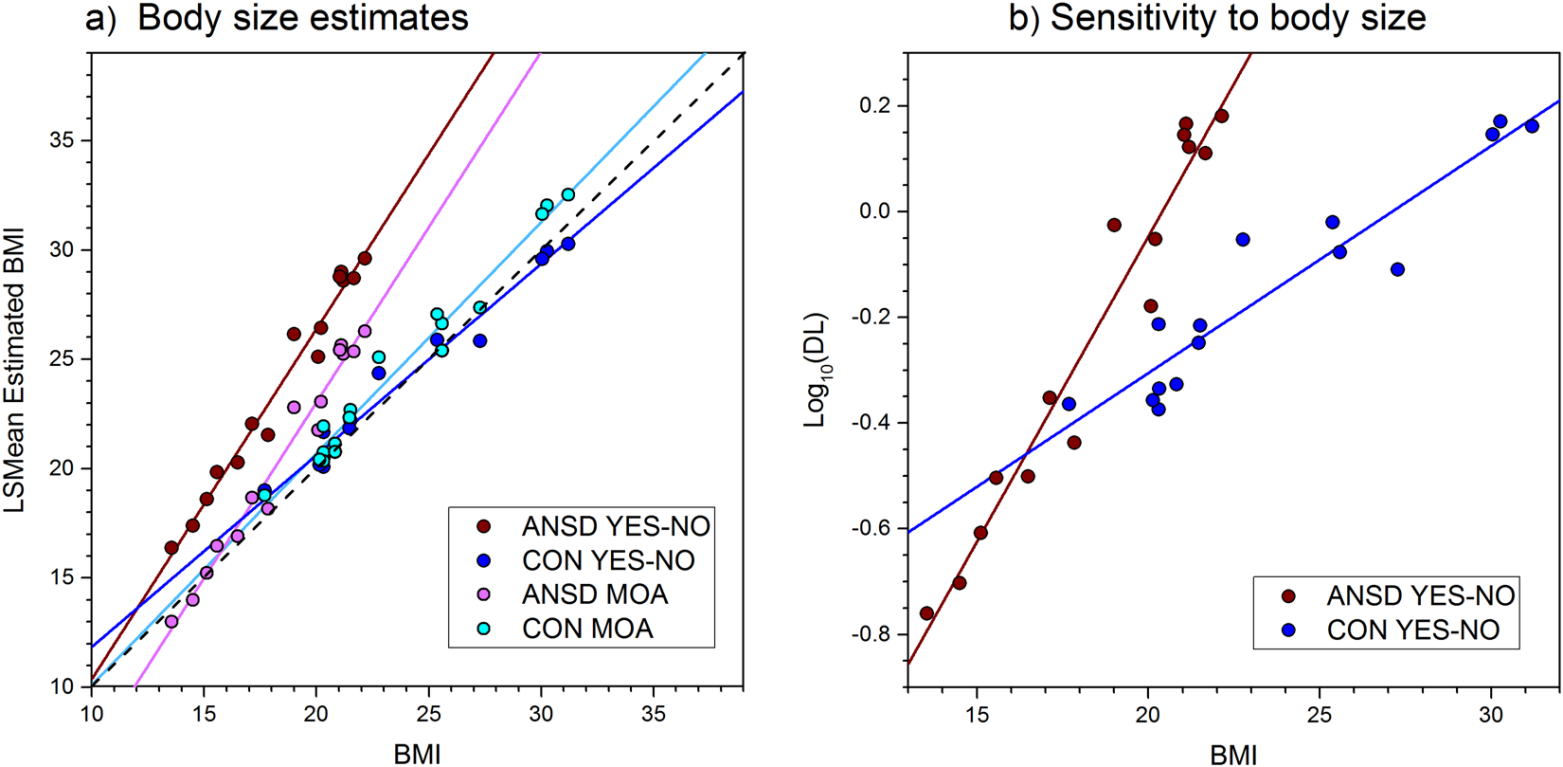
(**a**) Plots of estimated BMI as a function of actual BMI shown separately for: women with ANSD carrying out the yes-no task (brown data points with brown regression line) and Method of Adjustment (MOA) (pink data points with pink regression line) tasks, and for healthy controls carrying out the yes-no task (blue data points with blue regression line) and MOA (cyan data points with cyan regression line) tasks. The dashed black line represents the line of equivalence between estimated and actual BMI, corresponding to perfect accuracy in body size judgement. (**b**) Plots of Log10 DL as a function of actual BMI for the yes-no task, shown separately for women with ANSD (brown data points with brown regression line) and healthy controls (blue data points with blue regression line).

We used PROC MIXED (SAS v9.4, SAS Institute, North Carolina, USA) to build a generalized linear mixed model to predict participants’ estimates of BMI from their actual BMI, the GROUP to which they belonged (i.e. ANSD and CON, with CON as control) and the TASK they carried out (i.e. yes-no, using a standard model, and method of adjustment using avatars, with the method of adjustment as control) while controlling for the influence of PSYCH. The change in −2 Log Likelihood between the empty and final model corresponded to a statistically significant change in χ^2^(8) = 76.5, p < 0.001. The Type III tests of fixed effects for BMI, GROUP, TASK and PSYCH were: BMI, F(1,49) = 131.77, p < 0.001; GROUP, F(1,49) = 7.16, p = 0.01; TASK, F(1,49) = 0.62, p = 0.43; PSYCH, F(1,49) = 3.70, p = 0.06. In addition we found a statistically significant two-way interaction between BMI and GROUP, F(1,49) = 11.97, p < 0.001 and a three-way interaction between BMI and GROUP and TASK, F(2,49) = 4.80, p = 0.01. No other two-way interactions were statistically significant. We then used PROC REG (SAS v9.4, SAS Institute, North Carolina, USA) to test whether the slopes of the regression lines for estimated BMI on actual BMI, computed separately for each combination of GROUP and TASK while controlling for PSYCH, were significantly different from one: perfectly accurate performance in this task would correspond to a regression slope of 1 and an intercept of 0. The β weights for ANSD participants for the yes-no (β = 1.60, 95% CI = 1.44–1.76) and method of adjustment (β = 1.60, 95% CI = 1.45–1.76) tasks were both statistically significantly greater than 1 (F(1,13) = 67.63.4, p < 0.001 and F(1,13) = 42.50, p < 0.001, respectively). The β weight for healthy controls for the yes-no task (β = 0.87, 95% CI = 0.79–0.96) was statistically significantly less than 1 (F(1,13) = 8.80, p < 0.01). The β weight for healthy controls for the method of adjustment task (β = 1.05, 95% CI = 0.97–1.15) was not statistically significantly different from 1 (F(1,13) = 1.95, p > 0.2). The predicted values of estimated body size are plotted as a function of participant BMI in Fig. 3a.

Figure 3a shows that, having accounted for their psychological concerns about body shape, weight and eating, the regression slopes for women with ANSD were greater than 1 for both the yes-no and method of adjustment tasks (i.e. 1.6 and 1.6), consistent with the findings of Cornelissen *et al*.^32^. Specifically, women with ANSD who had the lowest BMI were the most accurate at body size estimation. As these participants’ BMI increased, so they rapidly started to overestimate their body size. By comparison, healthy controls who carried out the yes-no task showed a regression slope which was less than 1 (i.e. 0.87). In this group, those whose BMIs were closest to the population average of (i.e. ~26 for UK females^52,53^) estimated body size accurately, those with lower than average BMIs over-estimated and those with higher than average BMIs under-estimated. Therefore, healthy controls showed evidence for contraction bias when judging body size with the yes-no task (see also^32,34^). However, when healthy controls judged body size with the methods of adjustment task, the regression of estimated BMI on BMI had a slope almost exactly of 1 (i.e. 1.05). Therefore, it appears that whether the healthy control participants showed contraction bias for this kind of judgement depended on the experimental task they carried out.

Finally, Cornelissen *et al*.^32^ found that women with ANSD showed considerably greater sensitivity (i.e. smaller DL values) in the yes-no task than healthy controls at low BMI. However, as BMI increased, these authors found that task sensitivity for women with ANSD reduced much more quickly (i.e. a steeper rise in DL) than it did for healthy controls. Therefore, we used PROC MIXED (SAS v9.4, SAS Institute, North Carolina, USA) to test whether the same effects were replicated in the current data. The distributions of DL departed from normality (Shapiro Wilk’s W = 0.69, p < 0.001), and so were log transformed for further analysis. Both ANSD and control participants showed a positive, linear relationship between actual BMI and log10DL, F(1,22) = 14.83, p < 0.001. We found a marginally significant main effect of GROUP, F(1,22) = 3.37, p = 0.07, and a statistically significant interaction between GROUP and BMI, F(1,22) = 4.53, p < 0.05 showing that sensitivity in the yes-no task for women with ANSD is statistically different from that of the controls, as can be seen in Fig. 3b. Healthy control participants only showed a modest reduction in sensitivity to the task (i.e., increasing DL) with increasing BMI. However, this pattern was much more dramatic for participants with ANSD: low BMI participants with ANSD showed much smaller DL values than controls with similar BMI, suggesting very high sensitivity to the task. As the BMI of participants with ANSD increased, DL increased steeply towards the values of control participants, at a BMI of ~17. Finally, we did not find an effect of PSYCH, F(1,22) = 1.11, p = 0.30.

## Experiment 1 Discussion

The pattern of results for women with ANSD showed that the participants in this group who had the lowest BMI were most accurate at estimating their body size. As the BMI of ANSD participants increased, so did their over-estimation of their own body size, and this was true for both the yes-no task as well as the method of adjustment. Critically, this means that the results with the method of adjustment task replicate the findings of Cornelissen *et al*.^32^ with the yes-no task for women with ANSD. This therefore suggests that additional variance that we assume is introduced by asking women, who have differing underlying body shapes, to identify their perceived body size from a standard model, appears not to undermine the findings originally reported by Cornelissen *et al*.^32^. In short, there may therefore *not* be a body schema to stimulus mapping problem.

The healthy controls showed the pattern of estimation predicted by contraction bias for the yes-no task, but not for the method of adjustment task. Specifically, with the yes-no task, controls whose body weight is less than the population average tended to over-estimate their body size, those close to the population average were accurate and those whose body weight is higher than the population average tended to under-estimate. In comparison, performance of the healthy controls on the method of adjustment task was statistically equivalent to accurate body size estimation across the full BMI range. The most obvious reason for this difference lies in the fact that the yes-no task is not ‘anchored’, whereas the method of adjustment task is. Here, anchors comprise visual stimuli that consistently remind the participant about the smallest and largest body sizes in the stimulus range available to them. Such visual reminders were available to participants on every trial of the method of limits task, but not the yes-no task. The presence of task anchors can dramatically reduce or eliminate contraction bias^54^. However, this may not be a complete explanation, because there were two key differences between the yes-no task and the method of adjustment task: (i) the psychophysical procedure itself, and (ii) the standard CGI model seen by all participants in the yes-no task, versus the personalized avatars in the method of adjustment task. To disambiguate this situation, in Experiment 2 we recruited a sample of 60 healthy control women whose BMIs varied widely, and asked them to estimate their body size using the method of adjustment task, but this time with the same standard CGI model stimuli as was used with the yes-no task in Experiment 1. If anchoring effects cause the lack of contraction bias shown by the controls in the method of adjustment task in Experiment 1, then we should also expect to see no contraction bias in the method of adjustment task, even when the stimuli are derived from the standard CGI model.

The main rationale behind Experiment 2 was to demonstrate an absence of contraction bias with the method of adjustment task even with the standard CGI model. However, we reasoned that it would be useful to demonstrate a difference between this expectation and a second prediction using the same task, in the same participants with the same stimuli. Crossley *et al*.^55^ used CGI stimuli very much like those in the present study to ask women what body shape and size that they would ideally like. These authors found that, irrespective of their actual BMI, female observers selected ideal body shapes and sizes that centred on a BMI of 18–19. Therefore, in a second condition for Experiment 2, we asked participants to use the method of adjustment task to select the body size they would ideally like. In this way we expected to see a dissociation between the responses from the two conditions: what body size do you think you are (with no evidence of contraction bias) versus what body size would you ideally like to have.

## Experiment 2 Methods

### Participants

We recruited 60 female participants (chronological age M = 26.2, SD = 7.9) from the population of students and staff at Newcastle and Northumbria Universities and from the general population in and around the Newcastle upon Tyne area, all of whom gave informed consent to take part in this study. No participant had a history of eating disorders. Because we wanted only to measure psychophysical performance in these individuals in relation to their BMI, we did not administer any psychometric tasks. Data from one participant were omitted from the final analysis owing to a technical fault that prevented their responses from being recorded successfully.

### Psychophysical measurement procedures

#### Method of Adjustment tasks with standard model

Participants used the same method of adjustment task as in Experiment 1 to estimate their body size, but this time using the standard model stimuli (from the yes-no task in Experiment 1) instead of avatars, under each of two task conditions whose order was randomized across participants. Participants had to decide: the size and shape they believed themselves to have while viewing the whole body (BELIEF); the size and shape they would ideally like to have while viewing the whole body (IDEAL). For each condition, participants carried out 20 trials using the same display setup and procedure as for the Experiment 1.

## Experiment 2 Results

**Figure 4:**
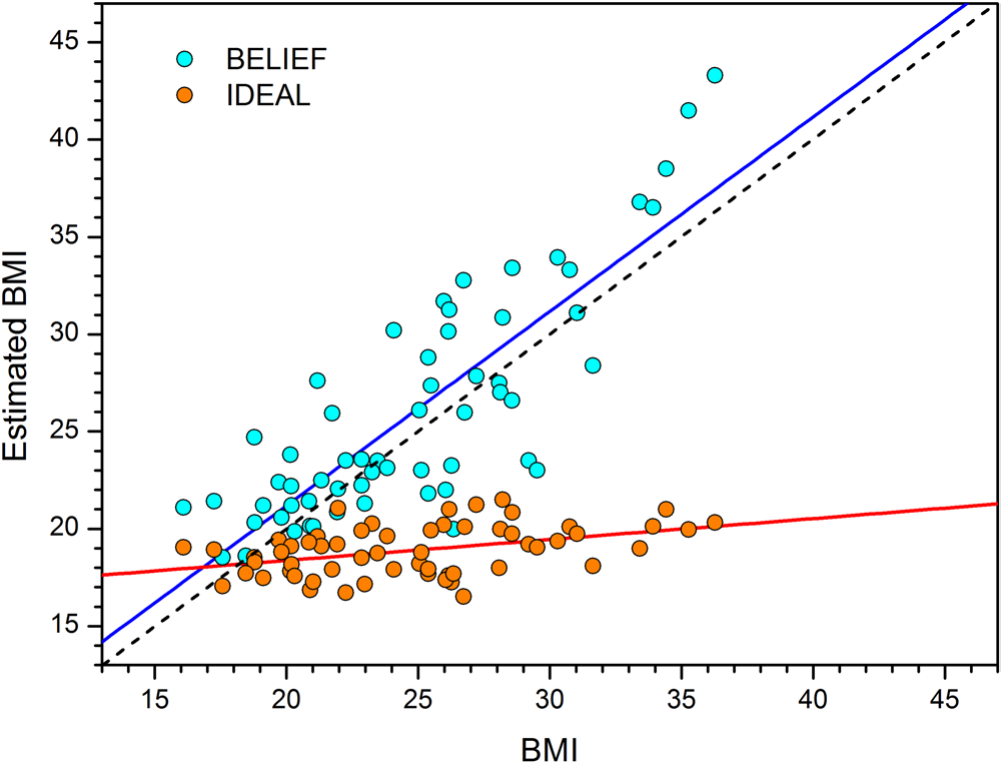
Plots of estimated BMI as a function of actual BMI from the method of adjustment (MOA) task shown separately for: healthy controls carrying out the BELIEF (cyan data points with blue regression line) and IDEAL (orange data points with red regression line) conditions. The black dashed line shows veridical performance with a slope of one and an intercept of zero.

Figure 4 shows plots of estimated BMI as a function of actual BMI for the 59 participants who carried out the method of adjustment tasks in Experiment 2 and for whom we had complete data. On inspection, it appears that participants accurately estimated their body size across the full BMI range. Conversely, it appears that irrespective of their actual body size, participants’ ideal body sizes fell within a very narrow BMI range, around 18–19.

These impressions were confirmed quantitatively using PROC REG in SAS v9.4 (SAS Institute, North Carolina, USA) to carry out ordinary least squares regression of estimated BMI on actual BMI, separately for the BELIEF and IDEAL judgements. The model for BELIEF estimates explained 69% of the variance in estimated BMI. The β weight for BMI was 0.99, t = 11.23, p < 0.001, and was not significantly different from a slope of 1 (F1,57 = 0.00, p = 0.99). In comparison, the model for IDEAL estimates explained 17% of the variance in estimated BMI. The β weight for BMI was 0.11, t = 3.40, p < 0.01, and was significantly different from a slope of 1 (F1,57 = 794.63, p < 0.001).

## Experiment 2 Discussion

The estimation of personal body size in Experiment 2 shows no contraction bias. There is no evidence of an over-estimation by observers with a low BMI or under-estimation by observers with a high BMI. Instead, as illustrated in Fig. 4, the estimates run parallel to, and just above the line of equality and show no BMI-related mis-estimation of size. As the participants are judging their own BMI, the estimates are naturally proportional to their BMI. By contrast, the participants’ estimates of their ideal body are not proportional to their own BMI, but cluster around a BMI of 18–19. This is consistent with a previous study using an interactive 3D program that allowed participants to create their ideal body, which reported an ideal BMI of 18–19 and which was not proportional to the participant’s own BMI^55^. It is also consistent with rating studies, which have asked participants to rate a set of photographs of women varying in BMI for attractiveness. Bodies with a BMI of 18–20 were rated as most attractive by both male and female participants’ irrespective of their own BMI^56,57^. These results demonstrate a clear dissociation between judgements of own body size and ideal body size.

**Figure 5:**
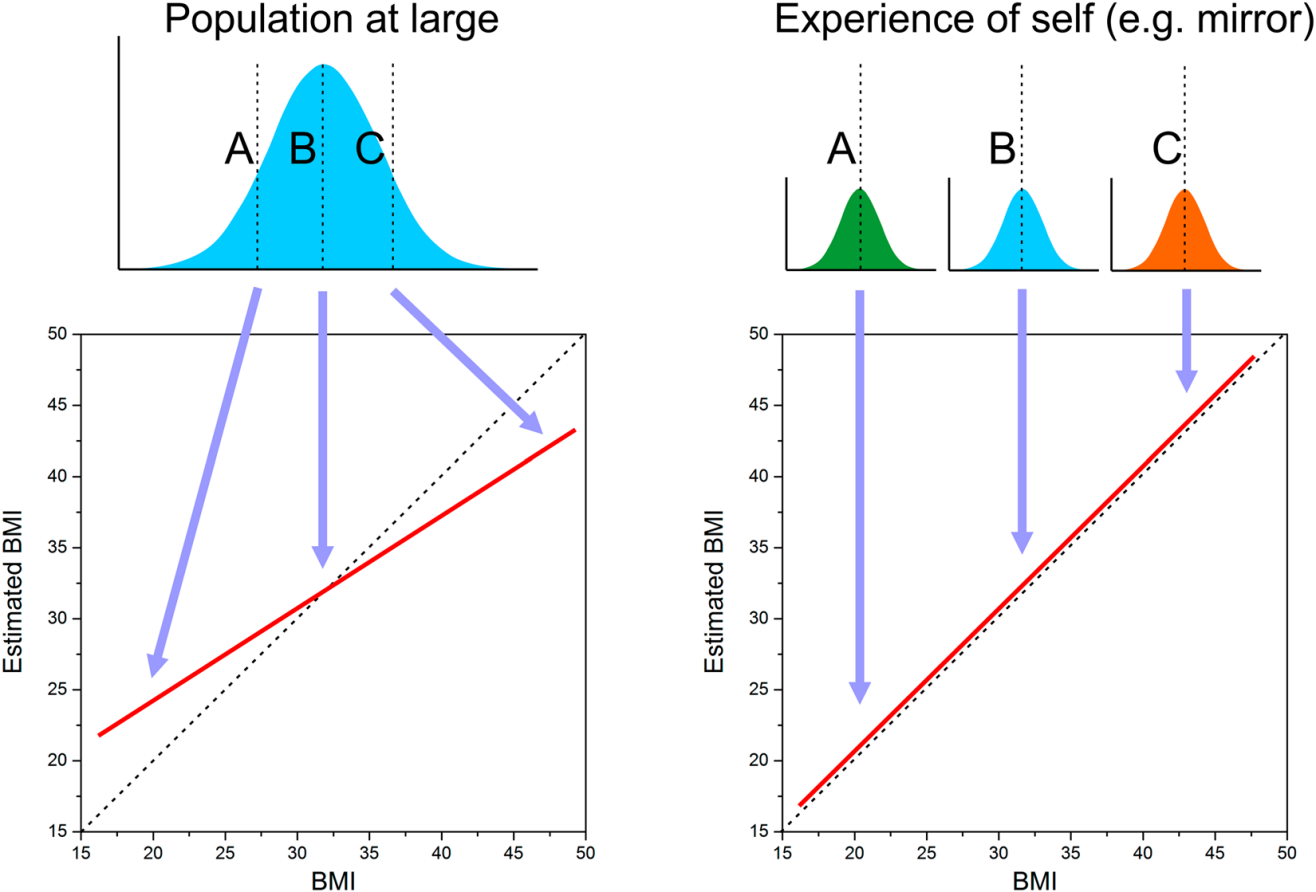
Illustration of the proposal that different body size distributions are used by observers as the ‘reference’ for an yes-no task (Population at large) and the method of adjustment task (Experience of self). **A**,**B** and **C** refer to three different individuals with a low, medium and high actual BMI, respectively.

When considering the results of the healthy controls in Experiment 1, although the most likely reason for the lack of contraction bias in the method of adjustment task was a potential anchoring effect, other possible explanations existed such as the use of a personalised avatar in the method of adjustment task and a standard body in the yes-no task. The results of Experiment 2 suggest that removal of these latter differences does not restore contraction bias to the method of adjustment task, and supports the idea that it is the anchoring effect that is responsible for the different pattern of results between the method of adjustment and the yes-no paradigms.

## General Discussion

For healthy control participants, the results of this study suggest that the accuracy of body size estimation is influenced by the paradigm being used to make the judgement. In the yes-no paradigm, control participants show a pattern of responses consistent with contraction bias (over-estimation of lower BMI bodies and an under-estimation of higher BMI bodies). This is a robust effect, and has previously been reported for judgements of own body size in yes-no paradigms^32^. In the method of adjustment paradigm, whether adjusting an avatar of the participant’s own body (Experiment 1) or a standard body (Experiment 2), contraction bias is absent. This seems to be due to an “anchoring effect”^54^. The anchors are the visual stimuli that show the participant the smallest and largest body sizes in the stimulus range available when the slider is adjusted. The abolition of contraction bias seems to be dependent on this link between movement of the slider and a direct change in the visual stimulus. For example, a participant may use a slider on a numerical range of 40 to 100 kg to indicate the weight of a stimulus body. If a slider is simply used to estimate the weight of a body that is fixed in size, the potential anchoring effects of knowing the maximum and minimum weight values that can be chosen, does not remove the contraction bias in the weight estimates^34^.

While we favour the “visual anchor” explanation for the lack of contraction bias with the method of adjustment task, there is another possible explanation, particularly with respect to Experiment 1. Previously, we have argued that the results reported from healthy control participants in a yes-no paradigm (with a standard model) may reflect their need to refer to a distribution of other peoples’ body sizes that they have learnt over their lifetimes, in order to judge whether the model they are looking at during the yes-no task is smaller or larger than they believe themselves to be^32,33^. In other words, this is exactly the situation that is required in order to observe contraction bias, where reference needs to be made by the observer to a learnt population norm (see left side of Fig. 5). However, in the method of adjustment paradigm in Experiment 1, the participant is trying to directly judge their own body size in relation to an avatar, a copy of themselves. In this case, the relevant reference distribution may well be the up-to-date memory of participants seeing their own body in the mirror, in videos, photographs and any other source of reflection. These distributions would therefore be normed with respect to each individual’s experience, and as a result not be subject to contraction bias, or at least minimally so, as illustrated on the right of Fig. 5.

The ANSD group in Experiment 1 showed a very different pattern of estimation. Both the yes-no and methods of adjustment tasks show that the ANSD participants are most accurate in their estimates of body size for the lowest BMI bodies, but as the ANSD individuals’ BMI increases, they systematically over-estimate their body size and the magnitude of this over-estimation scales linearly with their BMI. This is consistent with previous studies, which have shown a rapid increase in the degree of over-estimation as ANSD participant BMI increases^32,34^. One possibility for this pattern of judgements by ANSD women may be the development of an expertise effect in the judgement and discrimination of low BMI bodies. Women with ANSD spend a great deal of time looking at low BMI bodies including their own, but also online as part of their obsession with the thin ideal^58,59^. Repeated evaluation and discriminations of low BMI bodies could allow the development of an expertise in discriminating between low BMI bodies. Previous studies have suggested that practice in discriminating feature change can significantly improve the ability to make fine judgements; the expertise effect^60–62^. This expertise effect would be specific only to the low BMI bodies, as body shape changes in a non-linear fashion with increasing BMI^63^. Thus, the pattern of shape change with weight increase is different in low BMI bodies as compared to larger BMI bodies, and the expertise developed by women with ANSD would be specific to low BMI bodies and not generalise to judging higher BMI bodies

The pattern of DL results in Fig. 4, strongly suggests such an expertise effect. The results of the control participants show Weber’s law^64^. Weber’s law states that the just noticeable difference (JND) between two stimuli will be a constant proportion of their magnitude, leading to a constant Weber fraction over the stimulus range (i.e. ΔI/I = K, where I = stimulus magnitude and K = constant). This means that as a body gets heavier, it gets progressively harder to detect differences in body mass. This is the pattern displayed by the controls. By contrast, the ANSD women showed a finer discrimination at low BMI values than controls, but rapidly became worse than controls as the BMI of the bodies judged increases. The pattern of results suggests an expertise effect at low BMI values, but that this expertise does not generalise to higher BMI values explaining the rapidly increasing inaccuracy of the judgements as BMI rises. The fact that the ANSD women are focussed on judging very thin bodies, means that they lack practice in judging heavier bodies and so are actually worse than controls at these discriminations.

However, this is only a partial explanation. It does not explain why women with ANSD over-estimate body size rather than under-estimate it or instead just show a greater variability in estimates, but the same average accuracy of estimation as the controls (i.e. a greater random variation around the mean). Instead, the ANSD women show a rapid increase in the degree of over-estimation as the BMI of their bodies increases. This may result from their psychological concerns coupled with the documented aversion of individuals who have AN to making errors in judgements^65,66^. If one assumes that as the BMI of women with ANSD increases and their ability to make fine discriminations between body sizes reduces, the range of possible estimates they could make around the actual size increases. In the face of increased uncertainty, it may be their psychological concerns about their body size and an unwillingness to make mistakes, that pushes them to choose the higher BMI option of the possible BMI values.

An alternative conceptual framework to deal with this pattern of behaviour makes use of Social Comparison Theory^67^. If we assume that there is a cultural bias towards valuing the thin ideal in women’s body shapes (see^68^, then upward social comparisons (i.e. aspiring to the thin ideal) may lead to the experience of body dissatisfaction, and this may be particularly true for individuals who already have heightened concerns about body shape and weight. Thus, when such individuals are asked to pick which version of a standard model they think is closest to their own body size, they are willing to ‘pick’ an image which is larger than they are because this ‘cartoons’ their psychological distress. The degree of exaggeration or cartooning of their body size will increase as their actual body size increases beyond their ultra-thin ideal, reflecting their increasing body dissatisfaction.

This different pattern of results in the control and eating disordered groups with changes in the paradigm used to test them, illustrates a fundamental difference in the basis of the body judgements. The control group who are primarily making a perceptual judgement are impacted by the change in paradigm as this also modulates the strength of perceptual biases in the judgement. However, the women with ANSD show no difference in their judgements across techniques as their judgement is primarily determined by cognitive/psychological factors and so are less affected by the change in perceptual bias strength. This represents further evidence that body size over-estimation in women with ANSD is primarily based on cognitive/psychological factors^32^. This is a particularly important factor to consider in clinical practice as the over-estimation of body size rapidly rises as the BMI of the body they are judging rises and so as the patient’s weight rises in treatment, their over-estimation of their body size will show a disproportionate increase.

Of course, our study used only a relatively small group of women with ANSD. This new technique should be used in larger samples and extended to testing body image in men^69^. This methodology could also be used in other clinical groups who suffer from body image concerns, such as people with bulimia nervosa or people with body dysmorphia^70,71^.

The use of scanned bodies in body judgement paradigms suggests the interesting possibility of importing them into virtual reality (VR) environments. Previous studies have had volunteers interact with and even “inhabit” 3D bodies in a virtual reality environment^72–74^, but these are generic computer-generated bodies which are not biometrically calibrated for size and shape. Our study suggests that participants’ bodies could be scanned and imported into a VR environment, and they could then “inhabit” these bodies which can then undergo simulated weight change to produce a very realistic and immersive experience. This could be used both to measure body image disturbance and as part of an intervention in treatment. For example, the effectiveness of our own cognitive bias training program for body image which we have successfully piloted with women with AN in a conventional 2D format^75^, would potentially be more clinically effective in a VR format using scanned bodies.

This study demonstrates the viability of combining 3D scanning and CGI techniques to create personalised realistic avatars of individual patients to directly assess their body image perception. The absence of contraction bias with the method of adjustment suggests that it may be less prone to experimental artefact in the form of visual biases than other methods, such as the yes-no task, and may provide a clearer insight into the size and shape someone believes herself to be. However, this comes at the cost of not recovering the full psychometric function with the method of adjustment. The current results do not distinguish between a preference for personalized avatars over standard model stimuli. Nevertheless, we would argue the principle that in future studies we should move towards the most ecologically valid method for measuring self-evaluation of body size, i.e. the equivalent to looking in the mirror. Ultimately, the only way to achieve this is to use virtual reality technology to allow participants to inhabit a personalized 3D avatar in whom biometrically correct BMI dependent body shape changes can be manipulated by the participant in real time.
